# Modelling the age pattern of fertility: an individual-level approach

**DOI:** 10.1098/rsos.240366

**Published:** 2024-11-20

**Authors:** Daniel Ciganda, Nicolas Todd

**Affiliations:** ^1^ Max Planck Institute for Demographic Research, Rostock, Germany; ^2^ Instituto de Estadística, UDELAR, Montevideo, Uruguay; ^3^ UMR7206 ‘Eco Anthropologie’, Musée de l’Homme, CNRS, Paris, France

**Keywords:** agent-based modelling, fertility, reproductive process, demography, approximate Bayesian computation

## Abstract

Fitting statistical models to aggregate data is still the dominant approach in many demographic and biodemographic applications. Although these macro-level models have proven useful for a variety of tasks, they often have no demographic interpretation. Individual-level modelling, on the other hand, offers a deeper understanding of the mechanisms underlying observed patterns. Their parameters represent quantities in the real world, instead of pure mathematical abstractions. However, estimating these parameters using real-world data has remained a challenge. The approach we introduce in this article attempts to overcome this limitation. Using a likelihood-free inference technique, we show that it is possible to estimate the parameters of a simple but demographically interpretable individual-level model of the reproductive process by exclusively relying on the information contained in a set of age-specific fertility rates. By estimating individual-level models from widely available aggregate data, this approach can contribute to a better understanding of reproductive behaviour and its driving mechanisms, bridging the gap between individual-level and population-level processes. We illustrate our approach using data from three natural fertility populations.

## 1. Introduction

Modelling the age distribution of fertility rates is an essential step in a number of demographic applications. When only a tight fit to an observed schedule is required, as in the generation of single-age rates from grouped data, nonparametric models, typically based on splines, tend to produce the best results [1]. No particular price is then placed on whether each model parameter may be interpreted in any meaningful way. Other applications require models that can fit the data well while also providing a well-defined, ideally small set of parameters that represent quantities that can be interpreted in demographic terms [2–6].

The interpretability of parameters is particularly relevant in a forecasting context, where it may prove especially useful to associate a change in the value of the parameters with an underlying behavioural process, such as fertility postponement or the diffusion of contraceptive methods.

A difficulty associated with most existing parametric models is that the interpretation of their parameters can be elusive [7]. Even in the best cases, the relationship between mechanisms and parameters is ambiguous and indirect. In essence, macro-level models always operate one level above the processes that drive fertility.

A natural solution to this problem is to model at the individual level. This approach may be decomposed into three steps: (i) develop a micro model of the reproductive process, (ii) use this model to generate synthetic data and compute a set of simulated age-specific fertility rates (ASFRs) and (iii) estimate the parameters of the model by minimizing some measure of distance between simulated and observed rates.

The framework required for step (i) has long been developed by some of the pioneers of mathematical demography [8]. The ideas that emerged from this initial stage were later expanded to more realistic settings using simulation methods. The objective of this second wave of models was the incorporation of basic characteristics of the reproductive process such as age-dependent fecundability, or to represent change over time in fertility rates [9,10]. The use of simulation methods allowed researchers to complete step (ii), by generating synthetic ASFRs from individual-level models, drawing a direct connection between individual-level behaviour and observed, aggregate, quantities.

These contributions, however, remained largely theoretical. One key issue was that parameter values were borrowed from previous studies or calibrated through trial and error until the simulated data would appear to fit a given target distribution. In other words, estimation procedures remained too fuzzy, if not arbitrary, to decide whether individual-level models could be of use to analyse actual data.

Interest in these type of models dwindled after the 1980s. Ironically, the first statistical methods that would enable inference on complex simulation models started to emerge at around this time [11]. Likelihood-free inference methods, such as approximate Bayesian computation (ABC), have been extensively researched since then, providing a robust statistical framework to estimate the parameters of computational models [12]. In short, likelihood-free methods have reconciled statistical and mechanistic modelling by allowing the rigorous statistical estimation of sophisticated mechanistic models.

What we attempt to show in the remainder of this article is that these advances in statistical computing, that have proved so useful in fields such as ecology and population genetics, also provide the missing piece to the programme outlined above, finally completing step (iii). Specifically, these advances make it possible to infer individual-level quantities even when only aggregate fertility rates are available. More generally, the approach we advocate here provides a clear roadmap to fully integrate behavioural mechanisms in the modelling and forecasting of fertility trends.

To illustrate our approach, we use high-quality data from three natural fertility populations: Hutterite communities from 1860 to 1914, the French-Canadian population from 1700 to 1750 and a subsample of the Louis Henry Enquête in eighteenth-century France. The three datasets provide full maternity histories as well as information on the dates of other relevant events in a woman’s life course like marriage, death or death of spouse. However, we use the available individual-level data only for the validation of our results (see §4.3) and not for the estimation of our model because our objective is to show that individual-level quantities can be recovered when only aggregate data are available.

All the results presented in this article are fully reproducible using the code and data available at https://github.com/dciganda/individual_fx.

## 2. Model

The simplest form of the reproductive process is one in which the outcomes of the process (exact timing and number of births) are not a function of individual preferences. Louis Henry coined the term ‘natural fertility’ to refer to this type of process. A model of the reproductive process in a natural fertility setting requires three basic inputs: the moment when the process starts, typically marriage; the probability of a conception, known as fecundability [13]; and the length of the period in which a woman is not able to conceive following a childbirth, colloquially identified to post-partum amenorrhea.

We follow this approach and model the reproductive experience of a cohort of women to age 50 using a discrete-time simulation model, in which time advances at fixed increments of 1 month. The age (in months) at marriage for a woman i is simulated from a lognormal distribution, parametrized via its mean $\mu_{m}$ and standard deviation $\sigma_{m}$ .

Every month, married women who are neither pregnant nor amenorrheic are exposed to a probability of conceiving (her fecundability), which depends on her age. Whether susceptible woman i conceives in a given month when aged $x^{*}$ is the outcome of a Bernoulli trial with probability $\phi (x^{*})$ .

It is well established that fecundability progressively increases from puberty, reaches a peak during the early adult years and decreases progressively afterwards until menopause [14–16]. In order to capture this pattern, we model fecundability as a polynomial of degree 3. We define ϕ(x) as the fecundability at age x in the reproductive window (assumed 10–50 years), and $x_{s}=(x-10)/(50-10)$ , the age scaled to belong to [0,1] . Instead of using the standard basis $\left(1,x_{s},x_{s}^{2},x_{s}^{3}\right)$ , we use the Bernstein polynomials $B_{1}:x_{s}\mapsto 3x_{s}(1-x_{s}{)}^{2};\;B_{2}:x_{s}\mapsto 3x_{s}^{2}(1-x_{s});\;B_{3}:x_{s}\mapsto (1-x_{s}{)}^{3};\;B_{4}:x_{s}\mapsto x_{s}^{3}$ , decomposing ϕ(x) as $\sum_{j=1}^{4}\phi_{j}B_{j}(x_{s})$ . This enables us to naturally restrict our search to a subspace of dimension 2: since at age 10 ( $x_{s}=0$ ): $\phi (10)=\phi_{3}B_{3}(0)=\phi_{3}$ and at age 50 ( $x_{s}=1$ ): $\phi (50)=\phi_{4}B_{4}(1)=\phi_{4}$ , the fact that fecundability is known to be 0 at ages 10 and 50 immediately sets $\phi_{3}$ and $\phi_{4}$ to 0. What remains is therefore:


(2.1) $$\phi (x)=\phi_{1}\cdot B_{1}(x_{s})+\phi_{2}\cdot B_{2}(x_{s}).$$

**Figure 1.**
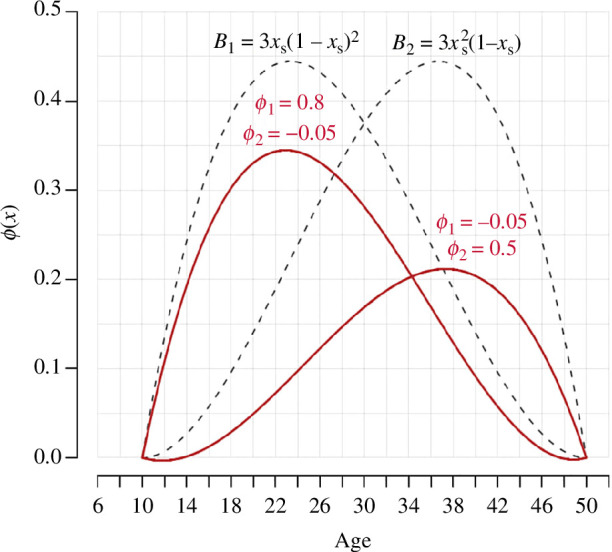
***Fecundability model.*** Although it depends on only two parameters, the model used to represent the evolution of the risk of conception across the reproductive life course can capture a wide range of patterns. The dashed lines represent the two Bernstein polynomials. The solid red lines represent different fecundability profiles associated with different combinations of $\phi_{1}$ and $\phi_{2}$ values.

Different combinations of $\phi_{1}$ and $\phi_{2}$ generate different age profiles for the risk of conception, as seen in figure 1. Given the age profile we expect, $\phi_{1}$ will have a stronger influence on the level at which fecundability peaks and $\phi_{2}$ will largely control the pace at which the risk of conception decays, until permanent sterility is reached. While the specific values of $\phi_{1}$ and $\phi_{2}$ are not informative on their own, the resulting age profile of fecundability produced by a given combination reflects both the behavioural (frequency of sexual intercourse) and biological mechanisms that influence the risk of conception.

A conception brings about a state of non-susceptibility (no risk of conception) equal to the duration of the pregnancy, 9 months, plus the duration of post-partum amenorrhea, δ , that must be estimated together with the other parameters of the model.

The model outputs individual reproductive trajectories, from which a set of simulated ASFRs ${,}_{s}f$ can be computed. We then leverage the distance between simulated and observed rates, ${,}_{o}f$ , to estimate the parameters of our model, namely $\left(\mu_{m},\sigma_{m},\phi_{1},\phi_{2},\delta \right)$ . This procedure is explained in more detail in §3.5, after we describe the data used in our analysis in the next section.

## 3. Data

### 3.1. Hutterites

The Hutterites are an Anabaptist community, originating in the fourteenth century in the Tyrolean Alps. After a long history of migration in Europe and Asia, they relocated to North America at the end of the nineteenth century where they still live today in self-sustained, largely isolated colonies.

Like other religious communities, the Hutterites oppose the use of birth control methods [17,18]. What made them stand out for demographic analysis was how strictly they adhered to these beliefs, at least until the second half of the twentieth century. The average marital fertility of the Hutterite cohorts born until the early 1900s remained around 10 children per woman, providing researchers with an exceptional opportunity to study the reproductive process under natural fertility conditions [19].

Another reason the Hutterites became the gold standard in natural fertility research was their practice of keeping detailed family records. These records, personally checked for consistency by colony preachers, were made available for various scientific studies in the 1950s and 1960s [19,20]. In the course of one of these studies, the original data were extended through follow-up interviews, which resulted in complete maternity histories for 562 families [21].

### 3.2. Historic Quebec

BALSAC is a longitudinal population database that contains information on individuals and families who have lived in Quebec from the seventeenth century to the present. The information used in this article, that concerns cohorts born before 1750, was gathered using family reconstitution methods from parish registers [22]. Given the high quality of these records, the information on seventeenth-century Quebec populations has become an important reference in the literature on natural fertility [15,23].

### 3.3. Historic France

Another important dataset for natural fertility research has been the Enquête Louis Henry. This dataset was created by Louis Henry and his co-workers in an effort to draw a nationally representative sample of the eighteenth-century French population. It contains data on life events for a sample of 378 parishes for the period 1670–1829 from parish and civil registers—the ‘anonymous sample’. Family reconstitutions were carried out for a subsample of 40 rural parishes—the ‘nominative sample’. For a detailed description of the Enquête, see [24]. We use the ‘nominative sample’.

A well-known problem in historical demography is that a small fraction of births may be missing from the parish and civil registers used. Specific procedures were developed by Henry to systematically assess and correct for these unregistered births [25]. Given our objectives here, we make no attempt to compute fertility rates corrected for these unregistered births. However, doing so would not pose any additional difficulties.

### 3.4. Sample sizes

To keep things simple, we model a process without death or union dissolution. Therefore, we restrict our data to ‘intact’ marriages, i.e. marriages that were neither lost to follow-up nor interrupted by the death of one of the spouses before the woman reached age 50. Although our model simulates the reproductive experience of a single birth cohort of women, we include information from multiple cohorts in the computation of our observed ASFRs in order to obtain reasonable sample sizes. To keep our hypotheses realistic, we analyse a range of birth cohorts that are as homogeneous as possible with respect to age at first marriage and total fertility rate (table 1).

**Table 1.** Sample information for the three natural fertility datasets.

	n**o**. **m**arriages	n**o**. **b**irths	**c**ohorts
Hutterites	161	1726	1860–1914
Historic Quebec	14 303	110 772	1700–1750
Historic France	3235	18 623	1680–1760

**Figure 2.**
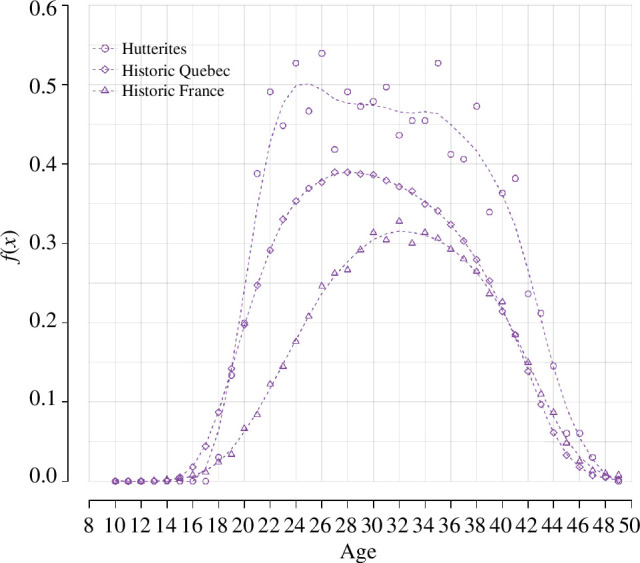
***Age-specific fertility rates for three natural fertility populations*** **.** Dashed lines show the fit of a cubic smoothing spline.

Figure 2 shows the observed ASFRs for our three natural fertility populations. As expected, the Hutterites show consistently higher fertility from age 20, with an average number of children per woman in these cohorts of 10.8. The French-Canadian cohorts have the second largest fertility in the three populations, with an average of 7.7 children per woman, followed by the French cohorts with an average of 5.7 children per woman. The distribution of ASFRs for the French cohorts also peaks at older ages, which is not surprising given their higher mean age at marriage, 25.8 years, compared with 20.6 years for Hutterites and 22.1 years for French-Canadian cohorts.

### 3.5. Estimation

As previously mentioned, our primary goal is to demonstrate that the lack of individual-level data is no impediment to understanding the behaviours and mechanisms underlying fertility processes.

When only aggregate data are available, the probability of individual observations cannot be calculated, and model parameters cannot be estimated using a maximum likelihood approach. Instead, we rely on ABC, a popular likelihood-free approach for the estimation of computational models.

The objective of ABC is to get a sample drawn from an approximation of the posterior distribution, without ever explicitly calculating the likelihood function. Instead, ABC algorithms simulate data from the model for a set of parameter values drawn from a prior distribution, compute summary statistics from each of these simulated datasets and keep only those parameter values that yield summary statistics that are close to those computed on the observed data, according to some predefined distance function. We naturally use the vector of ASFRs as our summary statistics and the Euclidean distance as our distance function.

The basic ABC rejection algorithm we use is straightforward to implement, but it requires a large number of simulations to obtain a sufficiently large sample from the posterior distribution [26,27]. One must accept keeping parameter values yielding simulated statistics only reasonably close to the observed statistics. To correct for this approximation and thus improve the efficiency of the method, one natural approach is to model the parameters as a function of the summary statistics in order to correct the parameter values that were accepted in the first step. This procedure, known in the ABC literature as ‘regression adjustment’, leads to a better approximation of the posterior distribution [28]. For this second step, we use a random forest (RF), as described in the pseudocode below.


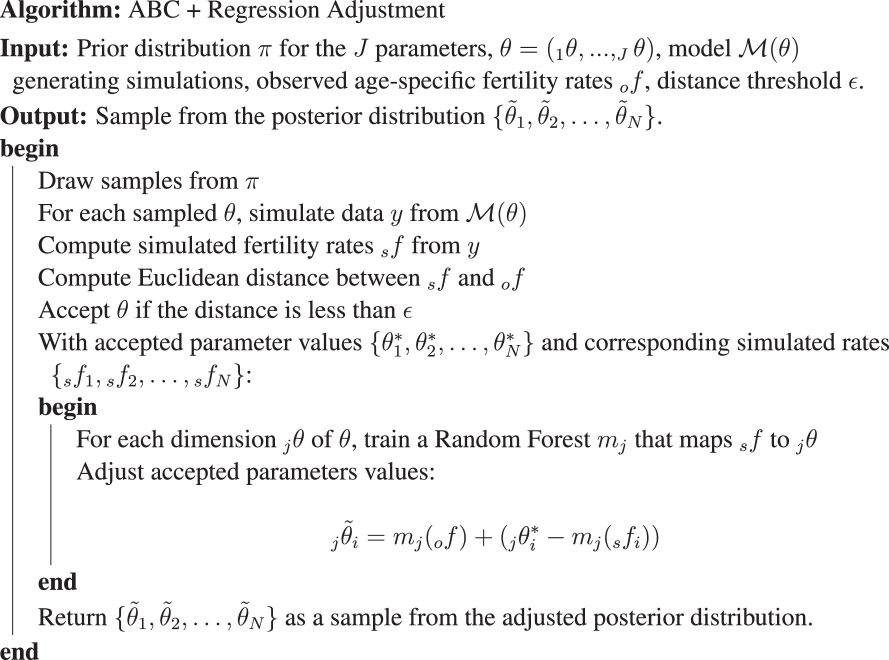


### 3.6. Sample size

As shown in table 1, the number of individual trajectories used to compute ASFRs varies widely between the three populations. Smaller sample sizes naturally produce noisier distributions of observed rates (as seen in figure 2) and therefore provide less information on the data generating process: having fewer data must result in a larger degree of posterior uncertainty about the parameters. To take this source of uncertainty into account, we match the number of marriages in our simulations to the number of marriages observed in each population, with the only exception of the French-Canadian sample. For sample sizes above 5000, the sample-to-sample variation in estimated fertility rates becomes extremely small: all vectors of rates simulated with 5000 marriages or more are similarly smooth. Since the computational cost of simulating the actual number of marriages in the French-Canadian sample (14 303) is prohibitive, in this case we simulate cohorts of 5000 marriages.

### 3.7. Prior choice

We use uniform priors for all our parameters and define a single prior distribution for the three populations analysed. In order to define lower and upper limits for these distributions, we reviewed the literature in a search for available estimates for our quantities of interest, as explained below. In all cases, this information was used to define a wide, plausible range of values for our search.

A compilation of estimates for peak effective fecundability (the maximum of function ϕ ) in natural fertility populations can be found in [29] and [30]. Taken together, these estimates range from about 0.15 to 0.30. We set the bounds of $\phi_{1}$ and $\phi_{2}$ to comfortably include these numbers, leading to peak fecundability values in the 0.12–0.35 range.

The duration of post-partum amenorrhea cannot be computed directly from maternity history information alone. However, a rough estimate of post-partum amenorrhea duration after the first birth can be obtained by substracting the mean duration between marriage and first births from the mean duration between first and second births. Given that the duration of non-susceptibility periods increases with age and parity, this procedure offers a good lower bound estimate for mean amenorrhea duration across the reproductive life course. Both [21] and [31] estimate this lower bound at 6 months in the case of the Hutterites. Leridon [29] provides estimates of 7.2, 8.6 and 10.2 months for three eighteenth-century French samples, which are not far from the estimate of 7.5 months we obtain by applying the method described above to the French sample (6.6 months for the French-Canadian sample). Taking these figures into account, we set the lower limit for the δ prior at 6 months and the upper limit at 18 months, which appears to be an upper bound for populations practicing extended breastfeeding [32].

Finally, we define the prior range for $\mu_{m}$ at ages 19–27 years, and for $\sigma_{m}$ at 2–7 years, both comfortably including the empirical means and standard deviations of the age at marriage distributions in our samples.

## 4. Results

### 4.1. Model validation

We use cross-validation to assess whether the parameters in our model can be correctly identified from the information contained in a set of ASFRs. For this, we randomly select the ith simulation as our validation set. The associated simulated rates are taken as pseudo-observed data. With all other simulations, the model parameters are then estimated as previously described, yielding a mean posterior ${,}_{j}{\tilde{\theta }}_{i}$ for parameter j . Repeating this procedure for 100 randomly chosen simulations, we compute the prediction error for parameter j as


$$ERR_{j}=\frac{\sum_{i}{(}_{j}{\tilde{\theta }}_{i}-\;j\theta_{i}{)}^{2}}{\text{Var}{(}_{j}\theta_{i})}.$$

**Figure 3.**
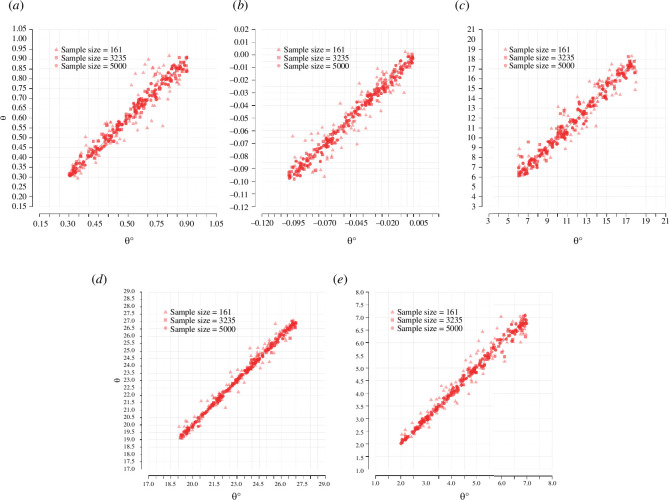
***Results of model cross-validation*** **.** The location of the red dots displayed in the figure is defined by the mean of the estimated posterior distribution in the *y*-axis, and the value of the parameter that generated the pseudo-observed data in the *x*-axis. (*a*) $\phi_{1}$ , (*b*) $\phi_{2}$ , (*c*) δ , (*d*) $\mu_{\text{m}}$ , (*e*) $\sigma_{\text{m}}$ .

The results of the cross-validation exercise are shown in figure 3. The three sets of results correspond to the different sample sizes used in the simulations. The results obtained with the largest sample size show that the estimation procedure consistently returns parameter values that are very close to the true parameters that generated the data. As expected, simulation results obtained with a smaller number of marriages are associated with large prediction errors.

Within a given sample size, the degree of uncertainty with which a parameter is estimated depends on to what extent the available data allow us to assign each region in the parameter space a distinct probability of having generated the data. When the effect of a change in a given parameter has an effect on the outcome that is similar to the effect associated with another subset of parameters, different regions of the parameter space will have similar probabilities of having generated the data, leading to wider posteriors.

Given this, it is not unexpected that the parameter with the smallest associated prediction error is $\mu_{m}$ and the parameter with the largest associated error is δ . Indeed, a change in the value of $\mu_{m}$ produces a shift of the entire distribution of rates that is very distinct from the effect of a value change in any other parameter. A change in the value of δ , on the other hand, produces an effect on the rates that overlaps to a certain extent with the effect of a change in the value of $\phi_{1}$ (see the electronic supplementary material for some animations illustrating the effect of each parameter on the schedule of ASFRs).

### 4.2. Model fit

**Figure 4.**
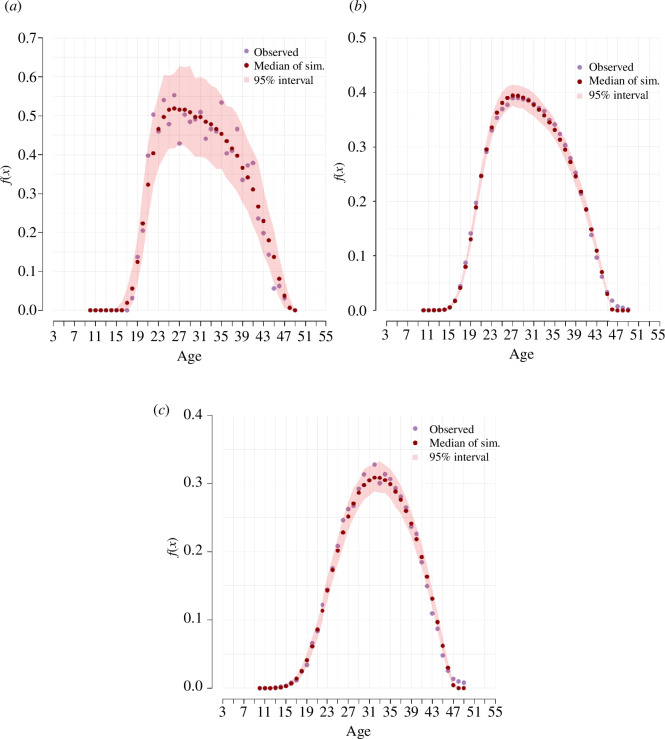
Model fit, 95% credible intervals. (*a*) Hutterites, (*b*) eighteenth-century Quebec, (*c*) seventeenth–eighteenth-century France.

Figure 4 depicts the model’s posterior predictive distribution against observed ASFRs for the three populations analysed. The model accurately captures the general characteristics of the data, such as the shape, location of peaks and rate of decline of the three distributions. In fact, the fit appears very satisfactory at most ages, except for the rates at the very end of the reproductive age window (ages 47–50), which the model tends to slightly underestimate. Credible intervals reflect the higher degree of uncertainty with which parameters are estimated in the Hutterites’ case.

### 4.3. Individual-level mechanisms

**Figure 5.**
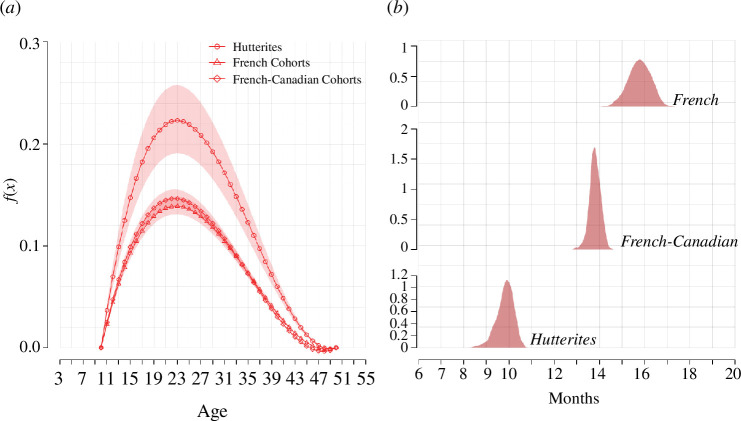
***Estimated fecundability and post-partum amenorrhea durations*** **.** The distributions of the probability of conception by age (*a*) were obtained by sampling a set of values for $\phi_{1}$ and $\phi_{2}$ from the posterior distribution (see appendix A) and applying equation (2.1). The lines represent the mean values of the resulting distributions and the shaded areas the quintiles that contain 95% of the distribution. (*b*) The posterior distributions for the length of post-partum amenorrhea in our three populations.

**Figure 6.**
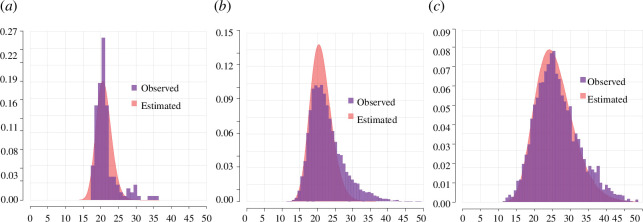
***Estimated versus observed age at marriage distributions*** **.** Shaded red areas represent densities for lognormal distributions with mean and standard deviation equal to the mean posterior values for $\mu_{m}$ and $\sigma_{m}$ (see Appendix A, figures 8–9). Purple bars represent observed ages at marriage in the three samples analysed. (*a*) Hutterites, (*b*) eighteenth-century Quebec, (*c*) seventeenth–eighteenth-century France.

Figure 5 shows the estimated age pattern of fecundability (figure 5*a*) and the estimated duration of post-partum amenorrhea for our three populations (figure 5*b*), while figure 6 displays the estimated versus observed age at marriage distributions. Taken together, they indicate that the higher ASFRs of the Hutterites are the result of a combination of higher fecundability, earlier marriage and shorter non-susceptibility periods. They also show that the model correctly explains timing differences in fertility in these populations not as a consequence of differences in the age pattern of fecundability, which is very similar in the three cases, but as a result of differences in marriage patterns.

Although the model captures well the highest density region of the age at marriage distribution, when the right tail of the distribution is more pronounced, as in the case of our French-Canadian sample, it tends to slightly underestimate its variance. The difficulties in correctly representing the right tail of these distributions are most likely related to the fact that we do not explicitly model any parity or marriage duration effects. By ignoring these effects, we are effectively computing the average fecundability and average length of post-partum amenorrhea over parity and marriage duration, making it harder for the estimation process to identify the exact proportion of marriages at each age. Nevertheless, the misalignment is relatively small considering the simplicity of the model and the data used for its estimation.

The peak fecundability that is implied by our estimates for $\phi_{1}$ and $\phi_{2}$ for the French and French-Canadian populations clearly falls on the lower end of the available estimates range (see §3.7). This is due to the fact that our model does not account for differences in couples’ conception probabilities. In the real world, couples are heterogeneous with respect to their fecundability. Given that the expected waiting time to conception for each couple is the inverse of its fecundability, the mean waiting time in the population as a whole is simply the harmonic mean of fecundability [29], on which couples with a very low fecundability exert a large influence. Thus, given an observed mean waiting time, not accounting for heterogeneity can lead to a fecundability estimate that is significantly lower than the mean fecundability effectively found in the population.

**Figure 7.**
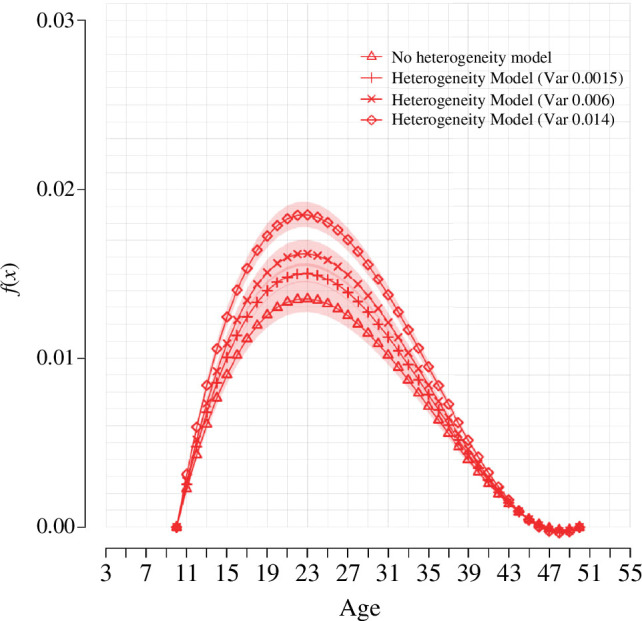
***Fecundability estimates. Model comparison for French cohorts*** **.** Triangles represent the estimated probability of conception at each age in the original model (as displayed in figure 5*a*). The rest of the curves show estimated fecundability by age for a model that accounts for differences in the probability of conception between women. Each line corresponds to an assumption about the variance of the distribution of fecundability at the age fecundability is at its peak. The shaded areas represent 95% prediction intervals.

Figure 7 compares fecundability as estimated with our original model with the estimates obtained with a model that accounts for heterogeneity (see below). As expected, the estimate for mean fecundability increases as the variance of the distribution of fecundability increases. Therefore, depending on the objectives of the analysis and characteristics of the data analysed, assumptions about the distribution of fecundability might be necessary.

To obtain the estimates under different variance assumptions, we model heterogeneity in fecundability assuming that each couple i has fecundability $\phi_{i}(x)=z_{i}\cdot \phi (x)$ , where z is Gamma distributed in the population: z∼Γ(s,s) . Setting the shape and rate equal ensures that E(z)=1 . Different heterogeneity scenarios can be obtained by fixing $\sigma^{2}=s^{-1}$ at different, credible, values. Estimating the model for each scenario then gives posterior medians for $\phi_{1}$ and $\phi_{2}$ . The variance values given in the legend of figure 7 are those for peak fecundability associated with these posterior median values, as variance at peak fecundability is the measure of heterogeneity that is most often found in the literature.

## 5. Discussion

The solutions at the aggregate level have predominated in dealing with the need of representing the age distribution of fertility rates. These solutions traded parameter interpretability in exchange for statistical formalization. Thanks to the approach we introduced in this article, this compromise is no longer necessary.

The individual-level model we introduce here is undeniably a simplistic, if not crude, description of the reproductive process. Nonetheless, it provides important insights into the behavioural and biological mechanisms underlying differences in ASFRs between populations, with minimal data requirements.

In particular, our results consistently show that it is possible to obtain a very good approximation of the timing of marriage for a cohort of women by exclusively relying on the information contained in a schedule of ASFRs. With respect to fecundability and the length of post-partum amenorrhea, the quality of the approximation will depend on the degree to which the assumptions implied in the structure of the model are met in the population analysed. In the case of fecundability, we showed that the estimation obtained depends on the variance of fecundability in the population analysed. In other words, as originally implemented, our model recovers the age pattern of fecundability implied by the data, under the assumption of homogeneous fecundability. When heterogeneity with respect to fecundability in the population of interest is high, or the objective of the analysis is to obtain precise fecundability estimates, our approach can be easily extended to incorporate assumptions about the variance of its distribution.

More broadly, our approach can be seen as a tool to transform a simpler representation of some data into a much richer representation of the same information. In our particular application, this new representation allowed us to understand differences in aggregate fertility rates in terms of differences in individual-level mechanisms across populations.

This approach is particularly useful in contexts where a process of interest is only observed through simpler, aggregate data. Such a scenario arises in biodemography, for example, when the demographic dynamics of wild animal populations are measured through census counts, instead of longitudinal monitoring data. For some of these populations, our model can be readily applied by simply adjusting the interpretation of age at marriage to age at the onset of reproduction.

The most obvious scenario, however, is contemporary human fertility, given that most vital statistics systems only collect count data on births. In fact, one of the objectives of our article is to spur the exploration of more complex models with the aim of understanding the microfoundations of present and future fertility patterns. These models will have to take into account the effect of individual preferences on the reproductive process, expressed both through the notion of a desired family size, as well as through the notion of short-term reproductive intentions that control the spacing of births across the reproductive lifespan.

The estimation of such models will very likely need additional information regarding, for example, the planned/unplanned nature of the observed births. In this regard, an important benefit of our approach is that such information can be incorporated as an additional target in the estimation procedure but can also be seamlessly integrated via prior distributions.

In light of the growing importance of data-driven, machine learning approaches in the study of natural and social systems, our emphasis on mechanisms may seem outdated. Ever larger amounts of data and computational resources combined with new algorithms seem to be shifting the balance in favour of pure-prediction versus explanatory approaches. However, as a number of recent studies suggest, the key to solving some of the most challenging problems in various disciplines may lie in a combination of mechanistic and machine learning approaches [33]. We hope the ideas presented here help bring together the mechanistic and statistical tools needed to further the study of demographic dynamics.

## Figures and Tables

**Figure 8.**
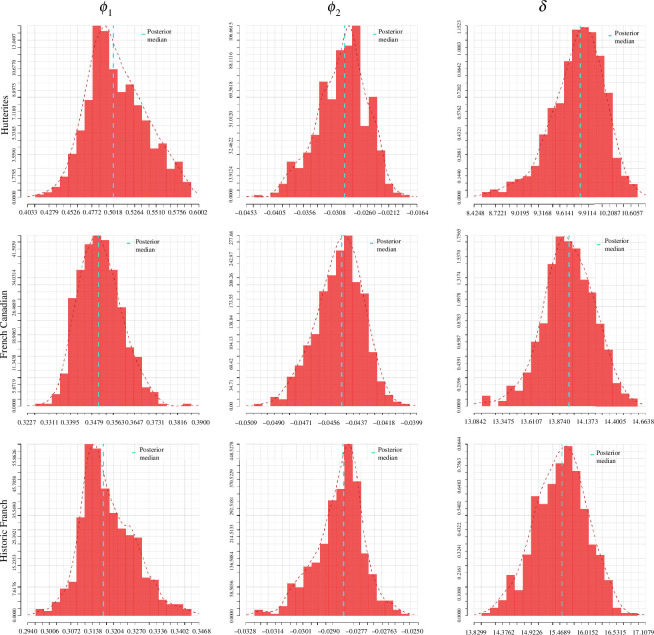
***Adjusted posterior distributions*** **.**

**Figure 9.**
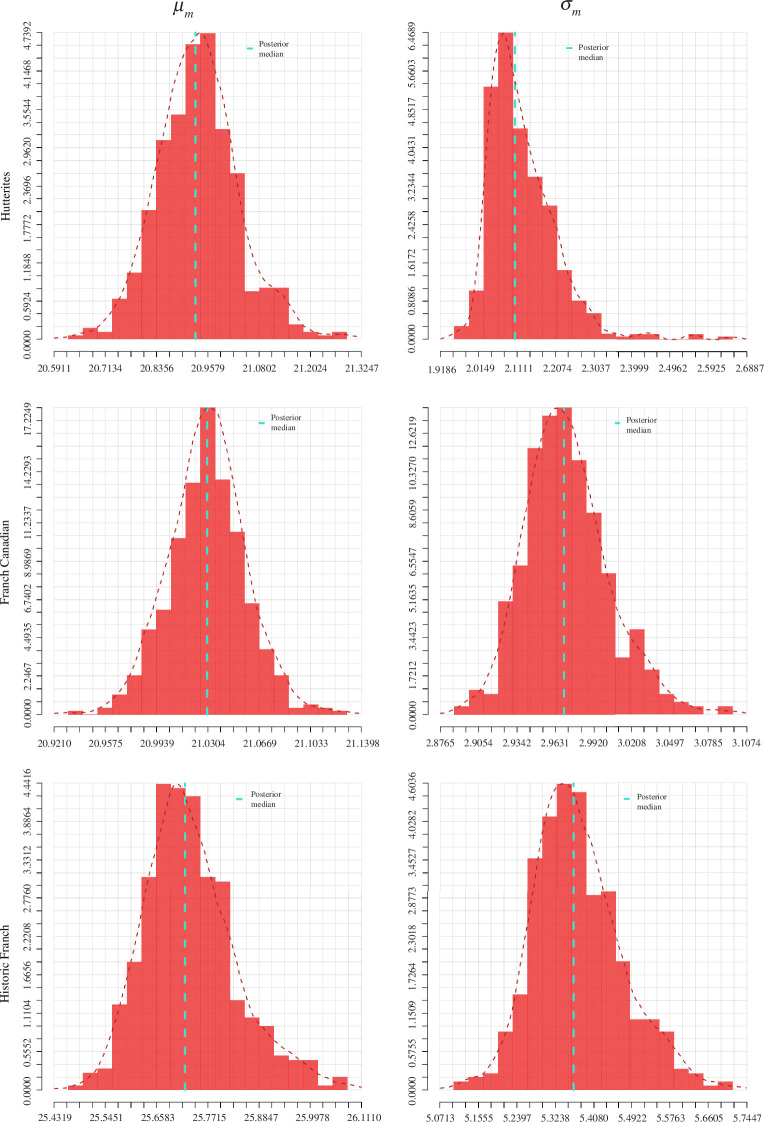
***Adjusted posterior distributions*** **.** Pink dashed lines show kernel density estimators for each sample.

## Data Availability

Data are available at GitHub [34]. Electronic supplementary material is available online [35].

## Appendix A

Figures 8 and 9.
